# P08 Audit of compliance with MHRA alerts with regards to patient counselling when prescribing fluoroquinolones

**DOI:** 10.1093/jacamr/dlaf118.015

**Published:** 2025-07-14

**Authors:** Lam Nguyen, Shazia Nazir

**Affiliations:** Leeds Teaching Hospitals (LTHT) NHS Trust, Leeds, UK; Leeds Teaching Hospitals (LTHT) NHS Trust, Leeds, UK

## Abstract

**Background:**

Fluoroquinolones (FQ) are a widely used broad-spectrum antibiotics that carry the risk of rare but irreversible adverse effects, including tendon rupture, suicidal thoughts, peripheral neuropathy, and central nervous system disorders. The Medicines and Healthcare products Regulatory Agency (MHRA) issued multiple alerts emphasizing the importance of patient counselling when prescribing FQ to ensure informed decision-making and mitigate potential harm.

**Objective:**

To assess fluoroquinolone counselling at LTHT per MHRA alerts and identify improvements in prescribing practice. This audit aims to assess whether patients at LTHT are being counselled about the risks associated with fluoroquinolones at the time of prescribing. By evaluating current prescribing practices, the audit aims to identify potential gaps and promote safer prescribing.

**Methods:**

Data collection began in March 2024 over two weeks, using the electronic prescribing tool to identify patients prescribed fluoroquinolones (ciprofloxacin, delafloxacin, levofloxacin, moxifloxacin, ofloxacin). *Inclusion criteria*: All clinical wards at LTHT including adults, paediatrics, and critical care. All patients prescribed any of the five fluoroquinolones specified above. *Exclusion criteria*: All patients taking fluoroquinolones for prophylaxis. *Data collection*: Patient demographic details (name, ward, age) and allergy status were collected. *Antibiotic prescribing*: The name, route, dose, indication, start date, and duration of FQ were recorded. Compliance with LTHT antimicrobial guidelines was assessed, along with whether input from an infection specialist or microbiology was provided. *Patient counselling*: Electronic records and drug charts were reviewed to assess whether patients were counselled before and/or after fluoroquinolone prescribing until discharge. Details of the healthcare professional providing the counselling and the method (verbal, MHRA leaflet, written) were recorded. If no counselling was documented, reasons were noted where available. *Risk factors and additional data*: Risk factors including age over 60 years old, concomitant use of corticosteroids, pre-existing psychiatric conditions and epilepsy were documented. Additional relevant information on dosing, reason for deviation from trust guideline, and side effects were also collected.

**Results:**

Sixty patients were reviewed and included in this audit. Counselling prior to fluoroquinolone initiation was documented for only 10 patients (17%), with the majority receiving an MHRA leaflet. 87% of patients were prescribed fluoroquinolones in line with Trust antimicrobial guidelines and/or microbiology input. One possible explanation for this adherence is that fluoroquinolones are first-line antibiotics in certain respiratory and urinary tract infection guidelines. However, despite following Trust guidelines, over 80% of patients did not receive counselling at the time of initiation, during treatment, or at discharge. Additionally, more than 75% of patients had at least one risk factor, with more than half being over the age of 60.

**Conclusions:**

The audit highlights a significant gap in adherence to MHRA alerts on fluoroquinolone counselling, with most patients receiving no documented counselling before treatment. Counselling remained remarkably low in high-risk patients including those over 60. This highlights the need for improved documentation and greater clinician awareness. The audit results have been shared in educational sessions and clinical governance forums to help raise awareness and encourage changes in practice. Moving forward, a re-audit is planned to assess improvements in prescribing practices.

